# Proteomic and microRNA data clarifying the effects of telomere shortening on cancer cells

**DOI:** 10.1016/j.dib.2014.12.003

**Published:** 2014-12-18

**Authors:** Orit Uziel, Meir Lahav

**Affiliations:** The Felsenstein Medical Research Center, Rabin Medical Center and Sackler School of Medicine, Tel Aviv University, Israel

## Abstract

In a previous study, we have shown that shortening of telomeres by telomerase inhibition sensitized cancer cells to cisplatinum, slower their migration, increased DNA damage and impaired DNA repair [1]. In the following study, we present a network model combining microRNA and proteomic profiling attempting to decipher the molecular mechanism underlying the effect of shortened telomeres on the obtained phenotype of cancer cells [2]. The microRNA and proteomic data were used for a network model construction, which provided us with several nodal candidates that may potentially mediate the shortened-telomeres dependent features. These protein expressions were experimentally validated, supporting their potential central role in this system [2]. In this article, we delineate the full proteomic data and a microarray analyses performed on cells with shortened telomeres compared to their cognate parental intact telomere cells. The data is attached as excel files. In principle, clarifying the mechanism behind telomere shortened phenotype may facilitate novel therapeutics development and may also obviate the time consuming process of telomere shortening achieved by telomerase inhibition.

**Specifications Table**

**Table:** 

Subject area	Biology
More specific subject area	The biology of telomeres and telomerase in cancer and aging
Type of data	Table
How data was acquired	Proteomic data was obtained by Quantitative mass spectroscopy (LTQ Orbitrap XL^™^ Hybrid Ion Trap- OrbitrapMass Spectrometer, Thermofisher).
	Microarray data were obtained by DNA microarrays hybridization (Affymetrix GeneChip^®^ Human Gene 1.0 ST arrays).
Data format	Raw
Experimental factors	Prior to the proteomic analysis, control cells were grown in the presence of SILAC (Stable Isotope Labeled Amino Acids) [3] and the experimental cells (in which telomerase was inhibited for 16 months exhibiting about 50% shortening) were grown in a standard growth media. For the microarray analysis, the control and experimental cells were grown in a standard growth media.
	Prior to the proteomic and microarray analyses telomerase inhibitor was withdrawn from the experimental media group to normalize for active telomerase in both cell groups (see Materials and Methods).
Experimental features	For the proteomic analysis cell cultures were grown for five population doublings as described above, lysed and proteins were extracted and separated on a 10% polyacrylamide gel. The mixed protein lane was divided into 10 pieces and subjected to quantitative mass spectrometry as described in [4].
	For the microarray analysis cells were grown as described above, RNA was extracted by the RNeasy mini kit (Qiagen, Hamburg, Germany) and measured by the Nanodrop (Thermo, Waltham, MA, USA), visualized on an agarose gel, aliquoted and hybridized to DNA microarrays as described in the Affymetrix website [5].
Data source location	Both analyses were conducted in Israel. The proteomic analysis was performed in The Smoler Protein Research Center, The Technion, Haifa. The microarrays analysis was done at The Genome Center in the Bioinformatic Unit, The George S. Wise Faculty of Life Sciences and the Sackler School of Medicine in Tel Aviv University, Ramat Aviv.
Data accessibility	The data is with this article.

**Value of the data**

• The issue of inhibiting telomerase as an anticancer target has been accelerated in the past decade. Many telomerase compound have been developed, some of them are already in clinical trials [6]. The inhibition of telomerase activity shortens telomeres and subsequently damage cancer cells. However, the mechanisms by which shortening of telomeres perturbs cancer cells are not fully elucidated yet. The data presented here including the study described in [2] is an initial step for understanding the mechanisms underlying cancer cells perturbation following telomeres shortening.
• Here we present a full proteomic data together with microarray analysis of cells with shortened telomeres versus the parental cells with intact telomeres. This data may be also valuable to researchers who conduct similar analyses for evaluation and comparison.

## 1 Data, experimental design, materials and methods

### 1.1 The experimental system

**Fig. 1:**
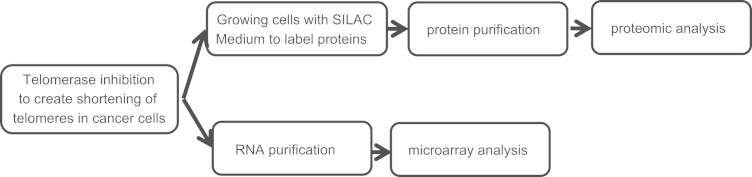
A flow chart of the study. See text for more detailed description of the experimental procedures.

SK-N-MC (neuro-epithelial neuroblastoma/Ewing sarcoma) cells were exposed twice a week to telomerase inhibitor, GRN163 (5 μM) for about 20 months (kindly donated by Dr. S. Gryaznov, Geron Corp. Menlo Park, CA, USA). The telomerase inhibitor was then withdrawn from the growth media and the cells were further grown for three additional population doublings to allow for elongation of the shortest telomeres and complete reconstitution of telomerase activity. The control intact cells were maintained in the culture medium without inhibitor. Telomere length was determined until shortening of more than 50% of the original length. Another set of control cells were created by withdrawing the inhibitor of telomerase from the growth medium until the telomeres reached their original size. See Fig. 1, the study flow chart.

### 1.2 The proteomic analysis

Cells were grown in the presence of SILAC-containing medium according to the protocol described by Mathias Mann, 2007 [3]. Basically, the control intact cells were grown in the presence isotope labeled L-arginine and L-lysine amino acids and the cells with shortened telomeres were maintained in a standard RPMI medium, containing “light” arginine and lysine. Both cell cultures were grown for five population doublings, lysed and proteins were extracted and separated on a 10% polyacrylamide gel. The mixed protein lane was divided into 10 pieces and subjected to quantitative mass spectrometry as described in [4]. The chosen cutoff for over or underexpression of proteins was twofold, after correcting the data to a 1:1 ratio. The proteomic data are summarized in Supplementary Table 1 A, B.

### 1.3 The microarray analysis

RNA was extracted from the above cells with the RNeasy mini kit (Qiagen, Hamburg, Germany) and measured by the Nanodrop (Thermo, Waltham, MA, USA), visualized on an agarose gel, aliquoted and hybridized to DNA microarrays (Affymetrix GeneChip^®^ Human Gene 1.0 ST arrays) as described in [5]. We used a total of eight chips in triplicates. Microarray analysis was performed on CEL files using Partek^®^ Genomics Suite TM, version 6.5 Copyright © 2010 [7]. Data were normalized and summarized with the robust multi-average method [8] followed by ANOVA. Gene expression data were sorted using cutoffs of *p*<0.05 under FDR (false discovery rate) adjustment criteria of *p*<0.0002 for NDF and of *p*<0.0005 for DF [9] and fold-difference cutoff of 1.5. The microarray data are summarized in Supplementary Tables 2 A (all genes) and 2 B (fold change – short telomeres versus intact telomeres).
